# The Interplay Between Affect, Dog's Physical Activity and Dog–Owner Relationship

**DOI:** 10.3389/fvets.2021.673407

**Published:** 2021-12-09

**Authors:** Heli Väätäjä, Päivi Majaranta, Anna Valldeoriola Cardó, Poika Isokoski, Sanni Somppi, Antti Vehkaoja, Outi Vainio, Veikko Surakka

**Affiliations:** ^1^Research Group for Emotions, Sociality, and Computing, Tampere University, Tampere, Finland; ^2^Master School, Lapland University of Applied Sciences, Rovaniemi, Finland; ^3^Department of Equine and Small Animal Medicine, University of Helsinki, Helsinki, Finland; ^4^Sensor Technology and Biomeasurements Group, Tampere University, Tampere, Finland

**Keywords:** dog, canine, physical activity, affect, emotion, activity tracker, welfare, dog-owner relationship

## Abstract

Leaving a dog home alone is part of everyday life for most dog owners. Previous research shows that dog–owner relationship has multifarious effects on dog behavior. However, little is known about the interplay between dog–owner relationship, physical activity of the dog, and affective experiences at the time of the owner leaving home and reunion when the owner comes home. In this paper, we explored how the general (daily, home alone, and over the 2-week study period) physical activity of the dog, and owner's perceptions of the dog's affective state were correlated at those particular moments. Nineteen volunteer dog owners had their dogs (*N* = 19) wear two activity trackers (ActiGraph wGT2X-GT and FitBark2) for 2 weeks 24 h/day. Prior to the 2-week continuous physical activity measurement period, the owners filled in questionnaires about the dog–owner relationship and the dog behavior. In daily questionnaires, owners described and assessed their own and their perception of the emotion-related experiences of their dog and behavior of the dog at the moment of separation and reunion. The results indicated that the dog–owner relationship has an interplay with the mean daily and weekly physical activity levels of the dog. An indication of strong emotional dog–owner relationship (especially related to the attentiveness of the dog, continuous companionship, and time spent together when relaxing) correlated positively with the mean daily activity levels of the dog during the first measurement week of the study. Results also suggest that the mean daily and over the 2-week measurement period physical activity of the dog correlated the affective experiences of the dog and owner as reported by the owner when the dog was left home alone. More research is needed to understand the interplay between affect, physical activity of the dog, dog–owner relationship, and the effects of these factors on, and their interplay with, the welfare of dogs.

## Introduction

Domestic dogs are highly social animals, and due to that, mutual relationships develop easily with humans (1). In Finland, as in many other cultures around the world (2, 3), domestic dogs are considered as family members, who usually live at the homes of their owners. The strong social bond between dogs and their owners resembles the attachment between a parent and a child, and includes characteristics present in a friendship [reviewed in (4)]. The bond between dog and human, therefore, resembles that of the attachment between humans, and the characteristics of the owner and the dog affect the relationship and, thus, also the behavior and interaction between the dog and the owner (1). However, the nature and level of the relationship varies (5). The close relationship has an important impact on the life of both the dog and the owner, especially at the emotional level. The dog–owner relationship affects the behavior of a dog (6, 7), and in turn, the behavior of the dog affects the attitudes of the owner about the dog relationship (8, 9), which may further affect the interpretations of the owner of the dog (10) and dog management practices (8). Usually, the quality of relationship is reflected on the behavior of the dogs particularly in situations where the dog is separated from its owner (6, 7).

The happiness and well-being of the dog is important to the owner. It is important for the owner to perceive the dog as happy as it also makes the owner happy (11). Taking care of the everyday needs and general well-being of the dog, such as ensuring adequate exercise and resting, contributes to the “happiness” of the dog as assessed by their owners (11). Leaving the dog alone at home is, however, something many dog owners worry about. To get more information about what happens when they are away, some owners have installed video cameras or pet remote monitoring systems in their homes, so that they can check on the dog and how it is doing (12). Also, solutions for remote communication by video calls and remote treat dispensers with video connection are on the market (e.g., Furbo Dog Camera). Monitoring dog activity and behavior can also be done by having the dog wear an activity tracker. From the activity trackers, the owners can check how much the dog has rested or been active generally while alone at home. Seeing the dog has mostly rested is comforting to the owner, while abnormal activity levels may alert about potential problems (11, 13). Owners can also make interpretations based on the behavior of the dog when leaving and returning home. Previous research shows that owners can be aware of separation-related behavior of the dog; this was confirmed by comparing video recordings with estimation of the behavior by the owner (14). In situations, such as when the dog is alone at home, the owner cannot monitor the behavior of the dog continuously. Activity trackers [e.g., (15, 16)], on the other hand, can measure the behavior of the dog continuously and could be potential complementary tools for assessing the behavior and affective states of the dog at home settings.

Dogs communicate flexibly by responding to human intentions and emotions, and showing their own. It is widely recognized that dogs have emotions, although at the moment, we cannot directly measure them, nor can we state how the animal subjectively experiences them as reviewed in Refs. (17, 18). Most of the previous research on dog emotions has used the discrete emotions (e.g., happy, sad, afraid) frame of reference as a way of assessing the affective states of the dog (17). The other way of looking at emotion-related behavior is the dimensional theory of emotions (19, 20). In this, typically, three bipolar dimensions (valence, arousal, and dominance) can be used for rating emotion-related experiences. The nine-point bipolar dimensions consist of (1) valence varying from unpleasant to pleasant, (2) arousal varying from deactivated (quiet, still, and relaxed) to activated (aroused), and (3) dominance varying from being in control to feeling of being controlled (i.e., potentially overwhelmed) by a situation or stimulus. The center of each scale reflects a neutral point, that is, neither unpleasant nor pleasant, for example. In human studies, the use of all three dimensions (valence, arousal, and dominance) is more common, whereas in animal studies, the focus has been on the two dimensions: valence and arousal. The physical activity of the dog may correlate to its arousal state (7, 21). Arousal can rise due to a positive (pleasant) or negative (unpleasant) emotion [(7); for a recent review, see (22)]. Although cognitive bias has been shown to be an indicator for animal emotion and welfare (23), and the dimensional approach to emotions has been shown to fit well in the assessment of the affective states of freely behaving dogs (24), dimensional approach has not yet been widely used in canine studies. Our research aimed to combine the information received from activity trackers with the subjective assessment and reporting of the owner of the behavior and emotions of the dog. It should be noted that in the dimensional theory of emotions, the dimension of dominance has a separate definition and meaning than in ethology (25).

In laboratory settings, the dog's emotions can be assessed by studying physiological measurements such as endocrine levels. For example, oxytocin levels rise, and cortisol levels decrease at the reunion of the dog and the owner (6). Heart rate and heart rate variability have also been used in assessing the emotions of the dog (24). In the natural settings of everyday life, emotions are typically assessed based on the behavior of the dog, by human observation or with the help of technology (15, 21, 24). Activity tracking can help in assessing how active the dog is throughout the day. Activity tracking is usually based on the measurement of three-dimensional acceleration. Measured movements can be categorized to different activity levels or classified to some activities (e.g., lying down, running) by signal processing algorithms [see, e.g., (26, 27)]. Following the dog's activity levels does not directly tell about the welfare or happiness of the dog. However, abnormal behavior and changes in the activity levels may indicate pain or some other issues affecting the well-being of the dog (28). When the dog is alone, the activity levels measured by activity trackers may indicate to the owner if the dog has been calm or restless (11, 13).

The aim of the study was to explore the interplay between the physical activity of the dog, dog–owner relationship, and affective experiences of the dog and owner measured with a three-dimensional model of emotions (valence, arousal, and dominance) both at the time of leaving the dog home alone and at the time of reunion. The study was conducted in everyday life of the participating dogs and their owners.

## Materials and Methods

The study was an exploratory field study. It was not controlled but was done “in the field,” in natural settings as part of the everyday life of the owner and the dog. The procedures of the field study were reviewed by the University of Helsinki Viikki Campus Research Ethics Committee (Statement February 2018, March 20, 2018) prior to the study. The human participants provided their written informed consent to participate in the study.

### Recruitment and the Criteria for Selection to Participate

Participants were recruited through a call for participation on the webpage and Facebook page of the project as well as through other local social media sites for dog owners in spring 2018. Pre-screening of volunteer owners was done based on their age (minimum 18 years), ownership of a smartphone using Android operating system, commitment to participate in the different phases of the data collection, and agreement to meet the researchers four times at set points in relation to the field study. In addition, the following dog-related criteria were used for choosing the participants: weight of the dog (10–50 kg), age (1–10 years), physical health, dog spending home alone at least 3 h on at least 4 days a week, being used to wearing a collar throughout the day as well as not being touch sensitive, so as to be able to wear the two activity sensors attached on the collar for the 2 weeks of the activity tracking measurements. Dogs with health-related and behavioral challenges, including separation-related problems, were excluded from the study. Decision to exclude these dogs was based on the fact that these issues may affect the activity of the dog either by increasing or decreasing the activity, and the health and behavioral problems were not in the focus of this exploratory study. Recruitment questionnaire also covered the daily living habitat of the dog, habits related to exercise, hobbies, information on household members and other dogs in the family, and the length of the time and weekly frequency that the dog spends home alone.

### Participants

Nineteen (*N* = 19) volunteer dog owners (17 females, 2 males, age: *M* = 33 years) were chosen to participate in the study. The most common breeds were Border Collie (3), Labrador Retriever (3), mixed breed (2), and the rest included, for example, Welsh Springer Spaniel, Golden Retriever, Nova Scotia Duck Tolling Retriever, Australian Shepherd, Doberman, and German Shepherd. Six of the dogs were males, and the rest (12) were females. Two males were neutered, four females were sterilized, and the rest were intact. The age of the dogs varied from 1 to 7 years (*M* = 4 years). All of the dogs were pet dogs, but most (except two) were also active in dog sports (obedience, agility, or similar), or in other hobbies such as search and rescue, breed-specific sports, and breeding or dog shows. All except one dog had two or more human family members (adult and/or child under 18 years). Eight dogs were the only dog in the household, whereas 11 participating dogs had 1 to 3 other dogs in the household. Most of the dogs lived in a suburban area (11), four in a city center, and three in a rural area. All dogs lived indoors. The total amount of daily exercise varied from 60 min to 4 h, depending on the household and the day of the week. None of the dogs was caged at home as this is forbidden by law in Finland, giving them a possibility to freely move within their homes at any time. All dogs, except one, were allowed free time off the leash during the exercise or in their own yard, for example.

### Activity Trackers and Activity Data Collection

The main features of the two activity trackers used in the study to collect data during the 2-week field study are presented in Table 1. Both sensors were attached to the collar of each participating dog for 14 days and collected data continuously over this period. Dogs wore the collar for the whole time, except while swimming, being washed, or participating hobbies or other activities not allowing the use of a collar or having sensors attached. Although two activity trackers were used to collect data, the results presented in this paper are based on the data collected with the ActiGraph® wGT3X-BT device. The data collected with this tracker was a complete three-axis accelerometer record recorded at 100 Hz. The Fitbark2® device gave access only to aggregate data computed over a longer period (1 min). Our original goal was also to compare the results obtained with different sensors; however, in this paper, we report results based on the Actigraph® sensor data. FitBark2® was chosen as a commercially available device for dog owners. It was needed in addition to ActiGraph® because it provided a mobile application for viewing the activity data and a Wi-Fi Base Station to wirelessly download the activity data during the research period. The Wi-Fi download also enabled the owner to view the activity of the dog remotely through the mobile application while not at home. After the recording period ended, the ActiGraph® devices and the FitBark® Wi-Fi Base Stations were collected from the participants and delivered to one of the authors. The author extracted the recorded Actigraph® data by connecting the Actigraph® device to a computer *via* a USB cable and using the ActiLife® software to initiate the download. For further processing with Python scripts, the data export feature of the ActiLife® software was used.

**Table 1:** Activity sensors used in the study.

**Device**	**Manufacturer**	**Weight**	**Sensors**	**Size**
ActiGraph wGT3X-BT	ActiGraph LLC, USA	19 g	Acc^*^	4.6 × 3.3 × 1.5 cm
Fitbark2	Fitbark Inc., USA	10 g	Acc (total)^**^	4.1 × 2.8 × 1.4 cm

* *Acc, 3D-accelerometer*;

** *Acc (total), 3D accelerometer readings taken multiple times per second and integrated over a 1-min epoch*.

### Field Study

**Figure 1:**
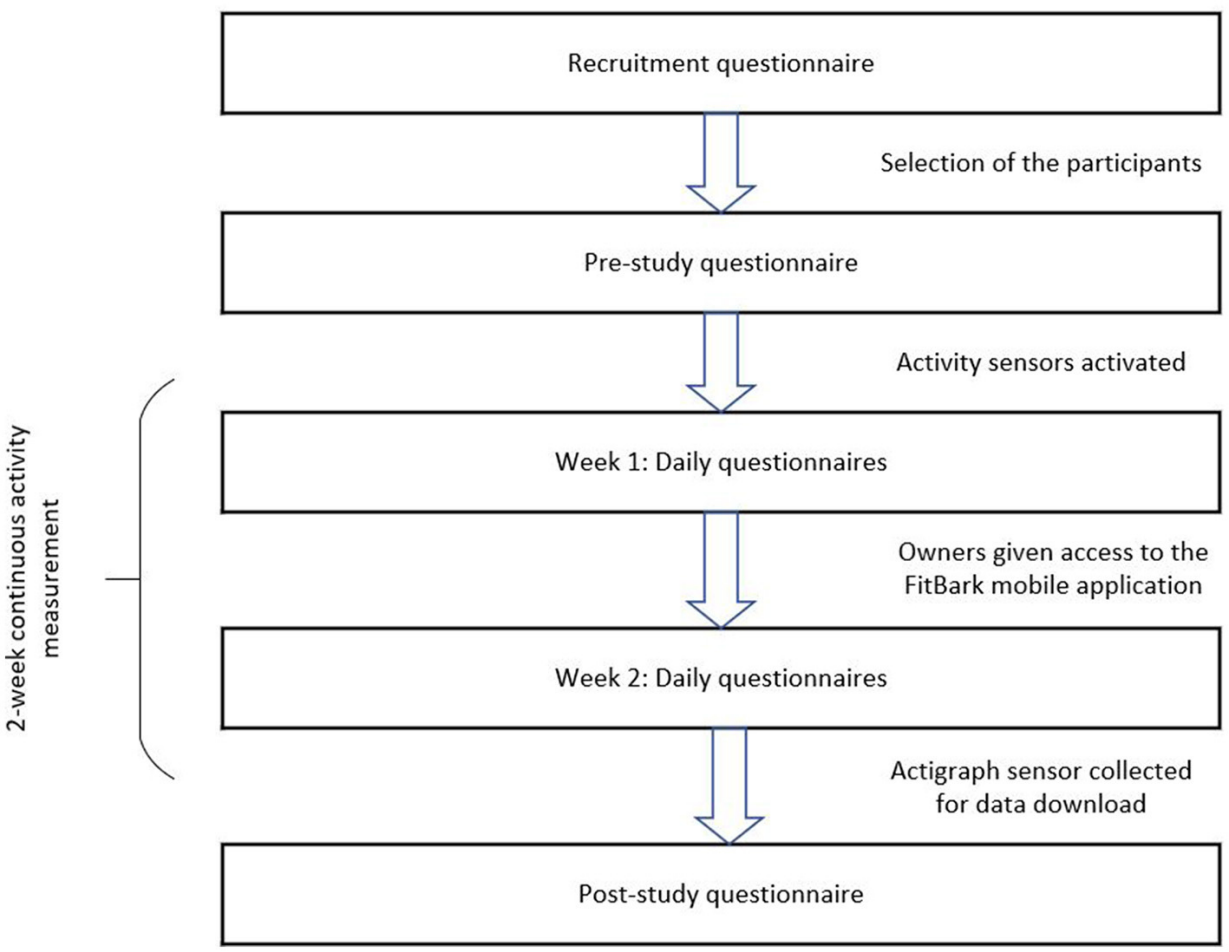
Data collection procedure and phases.

The 2-week field study was carried out in everyday life of the dogs and their owners from June to August 2018 at an appropriate time for the dog owner based on the availability and wishes of the owner. The field study consisted of the following data collection phases for each participant: a pre-study questionnaire, continuous activity data collection of the dog with the two activity trackers for the 2-week measurement period, daily questionnaires on affective experiences, dog behavior and activities, and a post-study questionnaire (see Figure 1).

After the selection of the participants based on the recruitment questionnaire, the owners of the selected dogs were sent an online pre-study questionnaire. The aim of the pre-study questionnaire was to collect information with open and closed questions on the daily routines, behavior, and habits of the dog, as well as departing- and rejoining-related routines and dog behavior at those moments as reported by the owners. In addition, pre-study questionnaire included validated scales to collect information on behavior and personality of the participating dogs using MCPQ-R [scales for extraversion, motivation, training focus, amicability, and neuroticism (29)] and C-BARQ [scales for separation-related behaviors and attachment and attention seeking (30)], and the human–dog relationship with the Monash Dog Owner Relationship Scale (MDORS) [scales for dog–owner interaction, emotional closeness, and perceived costs (31)]. The original scales in English were translated to Finnish first independently by four researchers, and a consensus on the Finnish translations for items of the translated scales to be used was made jointly by them in a meeting using translation back to English as well as discussion on the meaning of the items.

The 2-week (14 days) activity data collection started with a researcher visiting the dog owners at their home. First, the study phases and daily data collection with online questionnaires were explained to the owner as well as the opt-out option at any point of the study. After signing the informed consent, participants were instructed on the use of the scales for emotion ratings [(20)-see Attachment 1]]—instructions given to the owners for the meaning and use of the emotion dimensions and scales (see Attachment 2)—daily questionnaire: the three dimensional emotion measurement with nine-point scales). Then the two activated activity sensors were attached to the collar of the dog and a Fitbark2® sensor was connected to the wi-fi of the owners through the FitBark® Wi-Fi Base Station for data download. Activity sensor data collection was continuous during the whole 2-week period.

During the first week, the data collected with the Fitbark2® sensor was not visible to the owner. During the second week, the owners were given access to view the activity data through the Fitbark® mobile application. The aim was to explore whether being able to follow-up the activity of the dog would change, for example, the daily exercise routines. The daily online questionnaires covered the description of the activities and behavior of the dog in 4-h intervals throughout the whole day, the owner-reported assessment of the affective experiences of the dog, and the owner as well as the behavior of both at the time of departing and rejoining, when the dog was left home alone.

After the 2-week activity data collection, the ActiGraph® sensor and the FitBark® Wi-Fi station were collected by a researcher for data download. The participants were gifted the Fitbark2® activity sensor (value 40 dollars) as an incentive for the participation. A link to the online post-study questionnaire was sent to the owner for answering after ending the 2-week activity data collection. Figure 1 illustrates the data collection procedure and study phases.

### Analysis

The scales from MCPQ-R, MDORS, and C-BARQ questionnaires were first tested for internal consistency with Cronbach's alpha test. The results are reported in Table 2. The Cronbach's alpha analysis showed that these scales had an acceptable internal consistency, except from Attachment and Attention Seeking (C-BARQ). Correlations between the activity of the dog and MCPQ-R, MDORS, and C-BARQ scales, and the individual items from these scales were calculated with Pearson's correlation (two tailed). Spearman's correlation (two tailed) was used in calculating the correlations between the activity of the dog and the owner-reported affective experiences at the time of leaving and returning home when the dog was left alone at home. The mean daily, mean weekly, and mean activity of the dog over the 2-week period, as well as the activity during the time the dog was home alone based on the daily reported home alone times of the owner, were used in the statistical analysis.

**Table 2:** Reliability of the used scales from MCPQ-R, MDORS, and C-BARQ.

	**Cronbach's alpha**	**Number of items**
Separation-related behaviors (C-BARQ)	0.827	8
Attachment and attention seeking (C-BARQ)	0.301	6
Extraversion (MCPQ-R)	0.803	6
Motivation (MCPQ-R)	0.735	5
Amicability (MCPQ-R)	0.838	5
Neuroticism (MCPQ-R)	0.69	4
Dog–owner interaction (MDORS)	0.638	9
Emotional closeness (MDORS)	0.645	10
Perceived costs (MDORS)	0.801	9

*MDORS, Monash dog–owner relationship scale*.

Correlations between dog activity and signalment (weight, sex, and neutering status) were calculated, but no statistically significant correlations were found in this study. Information regarding the time when the collar was removed from the dog and time inside a moving car were extracted from the diaries kept by the owners and used to discard those periods from the data analysis. In addition, other information provided by the owners in the daily diaries was used to classify the remaining activity of the dog into different categories in addition to daily, weekly, and 2-week mean activity: activity outdoors, time alone, allowing access to/spending time in the yard, training, and others. Since minute-by-minute activity was not normally distributed, Wilcoxon signed rank tests were used to analyze it. First, a comparison of the activity between the 2 weeks was carried out with these tests to assess whether there was a difference in the activity, when the owner had access to the activity information. In this exploratory study, we did not have strong assumptions related to potential differences, but we were curious to see, for example, if owners who learn that the activity levels of their dog are low would react to this information by increasing activities. No indication was found for a change in the activity of the dogs between the 2 weeks.

Pearson's correlation coefficients were calculated between personality, dog–owner relationship, behavior, and activity of the dog, and Spearman's correlation was calculated between the activity of the dog and owner-reported dimensions of affect (valence, arousal, and dominance) at the time of leaving the dog home alone and at reunion. In the *Results* section, we focused on the results based on correlations on interplay between dog–owner relationship, dog activity levels, and affect that were significant or showed a trend.

## Results

### Dog–Owner Relationship and Physical Activity Levels of the Dog

Here we describe results based on Pearson's correlations (two tailed) found between the dog–owner relationship (from the pre-study questionnaire) and the activity levels of the dog (measured by the activity tracker) within the activity measurement period. It should be noted that no significant correlations were found between the whole scales related to dog–owner relationship and physical activity of the dog. Instead, there were some items of the scales that correlated with the physical activity of the dog.

The results indicate that the dog–owner relationship has an interplay with the activity levels of the dog. A strong emotional dog–owner relationship, as indicated by the following reported scale items, and the mean daily activity of the dog during the first week were significantly and moderately positively correlated. The more active the dog was (the higher the mean daily activity level during the first week of the field study), the higher was the owner-reported perception of the attachment of the dog to one person in a family (*r* = 0.526, *p* < 0.05, MDORS), attentiveness of the dog (*r* = 0.468, *p* < 0.05, MCPQR), continuous companionship provided by the dog (*r* = 0.518, *p* < 0.05, MDORS), as well as the time spent together when relaxing (*r* = 0.480, *p* < 0.05, MDORS).

### Physical Activity of the Dog and Owner-Reported Affective Experiences

Here we describe results from the Spearman's correlations (two tailed) between the daily questionnaires on the owner-reported affective experiences when leaving and when returning home, and the mean daily physical activity of the dog, mean physical activity over the 2-week period of the dog, and mean physical activity of the dog while home alone.

Results suggest that the mean physical activity daily of the dog and over the 2-week study period were correlated with the affective experiences of the dog as assessed by the owner, at the time of the owner leaving and returning home. The mean activity of the dogs over the whole 2-week study correlated significantly and slightly negatively with the ratings of the owner of the arousal of the dog at the time of being left alone, i.e., the more active the dog was, the more deactivated (relaxed, calm) the dog appeared at the moment of being left home alone (r_s_ = −0.218, *p* < 0.01). The mean activity of the dog over the 2-week study correlated significantly and moderately positively also with the ratings of valence (r_s_ = 0.336, *p* < 0.01) and significantly and slightly positively with arousal (r_s_ = 0.271, *p* < 0.01) when rejoining, i.e., the more active the dog generally was during the 2-week period, the more pleased (valence) and more activated or aroused (arousal) the dog appeared when the owner returned home. In addition, the mean daily activity during the 2-week study correlated significantly and moderately positively with the ratings of the owner of the valence of the dog (r_s_ = 0.361, *p* = < 0.01), and significantly and slightly positively with the ratings of the arousal of the dog (r_s_ = 0.256, *p* = < 0.01) when the owner returned after the dog had been alone at home.

There were also some correlations only present during the first week, during which the activity data were not shown to the owner, and during the second week, during which the owners could view the activity of the dog through the FitBark® mobile application. During the first week, the activity of the dog when home alone correlated significantly and slightly negatively with the own experience of the owner of being in control of the situation when leaving home (r_s_ = −0.281, *p* = < 0.05), i.e., the higher the activity when the dog was alone, the lower was the own experience of the owner of being in control (i.e., feeling overwhelmed) in the situation when leaving home. Also, during the first week of the study, the activity of the dog while home alone correlated significantly and slightly negatively with the owner-reported valence of the dog when returning home (r_s_ = 0.237, *p* = < 0.05), i.e., the higher the activity while home alone, the lower the rating of the owner of the valence of the dog while the owner returned home. During the second week, the activity of the dog when home alone correlated significantly and moderately negatively with the ratings of valence of the dog at the time of the owner leaving home, i.e., the higher the mean activity of the dog was when the dog was alone at home, the lower were the ratings of the owner for the valence of the dog when leaving (r_s_ = −0.313, *p* < 0.01). The activity of the dog while home alone correlated significantly and moderately positively with the own arousal of the owner when returning home (r_s_ = 0.319, *p* < 0.01) during the second week. Whether being able to monitor the activity of the dog remotely from the FitBark® mobile application during the second week affected the affective experiences of the owners, and possibly their behavior as well as the ratings of affective experiences at the time of returning home, cannot be concluded from this study.

## Discussion

Our findings showed some positive associations between activity measurements, questionnaire, and rating scale data. Although the correlations were significant, the coefficients for determination (i.e., squared value of correlations) were relatively small. As usual, correlation does not imply causation. That is, we do not know why these things were correlated and cannot conclude that the behaviors of the owner and the dog, activity of the dog, affective experiences, and dog–owner relationship influenced each other in a specific way. However, in the interest of finding useful hypotheses for experimental follow-up studies, it is worthwhile to discuss the phenomena that could have led to these correlations and might influence the outcome in dog–owner interactions in general.

### Dog–Owner Relationship and Physical Activity Levels

The positive correlation between dog–owner relationship and mean daily activity levels found in this study is in line with previous research; for example, the strength of the dog–owner relationship is correlated with dog walking (32). Influence of physical activity with a possible positive effect on dog behavior and reducing undesirable behaviors has been reported (33), and may influence the human–dog relationship as perceived by the owner. Furthermore, more frequent owner interaction and participation in activities and training has been associated with less fearfulness of pet dogs (34), which may be experienced also as a better human–dog relationship.

The daily reports of the owners from our study indicate that active owners spend a lot of time and engage in many activities (e.g., agility), with their dogs. The time spent together increased the activity levels, and time spent together can also further improve the emotional attachment. It should be noted that the activity tracker attached to the collar also measures head movements; thus, attentively following what is happening around may increase the low-level activity. In this study, the different types of movements were not categorized (in analysis with ActiGraph®, although some categorization was shown to the participants in the FitBark® mobile application). The participants in this study were relatively active, which might bias the results or at least not allow full extrapolation to the general population.

### Physical Activity Levels of the Dog and Affective Experiences When Left Home Alone

Our results showed that active dogs were calmer (i.e., less aroused) when being left alone. As previously discussed, research suggests that physical activity can have a positive effect on dog behavior and reduce fearfulness and undesirable behaviors (33, 34). On the other hand, valence was rated as being more positive and arousal level was higher for dogs with more physical activity, when the owner returned home, suggesting a happy behavior at the reunion. It has been suggested that a more enthusiastic greeting behavior may suggest that being alone evokes higher negative affective states, indicating insecurity while being left home alone (14). If the dog is insecure, it can be assumed that the valence is reported by the owner as more negative, possibly combined with high arousal, and the dog may be overwhelmed by the situation when rejoining after separation. However, as the owners of the dogs with higher physical activity reported in our study more positive valence combined with higher arousal at the time of reunion, it seems that this behavior is happy. On the other hand, if a dog is not active to greet the owner happily, this could indicate a problem also in the human–dog relationship.

Interestingly, when owners reported being less in control of the situation when leaving home during the first study week, the activity levels of the dog while being alone were higher than when the owner reported being more in control when leaving the dog home alone. This can indicate, as the open question results in daily questionnaires somewhat revealed, that the dog reflects either the affective state or behavior of the owner and/or when something is out of the ordinary, the dog is more easily distressed and restless when left alone. However, there may be other affecting factors at play, like changes in exercise prior to being left alone, etc., that this study and data do not reveal. Furthermore, the higher the activity when alone correlated with the lower (more negative) valence as reported by the owner at the time of separation in the second week.

It should be kept in mind that the results related to affective experiences are based on the subjective perception of the owner of the situation. The own emotions and assumptions of the owner may affect how they perceive the situation and how they interpret the behavior of the dog. According to responses to the open questions of daily online questionnaires, the owners clearly wanted to understand their dog and tried to organize their daily routines so that the time alone would be as easy as possible for the dog (e.g., by giving treats when leaving, or keeping the moment of leaving calm). Observing the stress of the dog also seemed to make the owner feel stressed, and the other way around: the dog may have reacted to the stress or arousal of the owner.

### Limitations of the Study

The number of participants in this study was fairly small. It is also likely that active dog owners who have strong emotional relationships with their dogs and who are interested in the welfare of their dogs will volunteer in this kind of study. We also decided to exclude dogs with health issues and behavioral problems from the study to minimize the effect of these issues on the data collection with activity trackers, as the effect could be potentially either increasing or decreasing activity. Thus, dogs and owners participating in the study represent a limited view of the whole dog–owner population. The study was not controlled but was done in natural settings as part of everyday life of the owner and the dog. The daily activities of the dogs and the owners varied within and between participants over the 2-week study period. During the study, Finland happened to have an abnormal heatwave, which may have affected some of the typical daily activities for some participants (e.g., a participant reported replacing walking by swimming and taking the activity tracker away during swimming). There were variations and inaccuracies in the daily diaries, making it sometimes hard to decide, for example, if some data should have been excluded or not, or connecting the descriptions of the owner with the measured data. Without video recordings, it is impossible to say how accurate and complete the descriptions, interpretations, and assessments of the owner of the emotions and behavior of the dog were.

## Conclusion

The results suggest a possible link between the dog–owner relationship, physical activity levels of the dog, and affective experiences of the dog. A strong emotional dog–owner relationship correlated positively with the mean daily activity levels of the dog during the first week. The daily and daytime activity levels of the dog were related to the affective experiences of the dog and owner when the dog was left home alone. More research is needed to understand the interplay between affect, physical activity of the dog, dog–owner relationship, and the effects and interplay of these factors on the welfare of the dog. Our study also suggests that the dimensional model of emotions can be a useful tool in studying the affective experiences of pet dogs and may hold potential in assessing the affective states in relation to welfare of the dogs. The results also demonstrate the types of possibilities that relatively simple technology, such as activity trackers, may provide to support the strengthening of the dog–owner relationship through better understanding of own dog and affective states of the dog. In addition, the results illustrate opportunities to develop and use low-cost tools for following up and exploring the welfare aspects and effects of different issues in everyday life on dog behavior and wellbeing.

## Data Availability Statement

The raw data supporting the conclusions of this article will be made available by the authors, without undue reservation.

## Ethics Statement

The animal study was reviewed and approved by University of Helsinki Viikki Campus Research Ethics Committee, Finland. Written informed consent was obtained from the owners for the participation of their animals in this study.

## Author Contributions

HV designed the study, performed the data collection, participated in the preparation of the data and data analysis, and participated in the manuscript preparation. PM participated in the planning of the study and in the data analysis, participated in the manuscript preparation, and participated in acquiring the funding. AC participated in the preparation of the data and data analysis and participated in the manuscript preparation. PI participated in the planning of the study and data collection, preparation of the data, and manuscript review. SS participated in the planning of the study, and preparation of the manuscript. AV and OV participated in acquiring the funding and in the manuscript review. VS supervised and administrated the project at Tampere University, and participated in acquiring the funding, the planning of the study, the manuscript preparation, and the review. All authors contributed to the article and approved the submitted version.

## Funding

This research was funded by Business Finland in the Turre ja Toivoset 2.0 project (The Buddy and the Smiths 2.0; grants 1665/31/2016, 1894/31/2016, 7244/31/2016).

## Conflict of Interest

The authors declare that the research was conducted in the absence of any commercial or financial relationships that could be construed as a potential conflict of interest.

## Publisher's Note

All claims expressed in this article are solely those of the authors and do not necessarily represent those of their affiliated organizations, or those of the publisher, the editors and the reviewers. Any product that may be evaluated in this article, or claim that may be made by its manufacturer, is not guaranteed or endorsed by the publisher.
